# The oncogenic potentials and diagnostic significance of long non‐coding RNA LINC00310 in breast cancer

**DOI:** 10.1111/jcmm.13750

**Published:** 2018-07-11

**Authors:** Juan Li, Wanxin Peng, Lutao Du, Qifeng Yang, Chuanxin Wang, Yin‐Yuan Mo

**Affiliations:** ^1^ Department of Clinical Laboratory The Second Hospital of Shandong University Jinan Shandong Province China; ^2^ Cancer Institute University of Mississippi Medical Center Jackson MS USA; ^3^ Department of Cell biology School of Medicine Jiangsu University Zhenjiang China; ^4^ Department of Breast Surgery Qilu Hospital Shandong University Jinan Shandong Province China; ^5^ Pathology Tissue Bank Qilu Hospital Shandong University Jinan Shandong Province China; ^6^ Department of Pharmacology/Toxicology University of Mississippi Medical Center Jackson MS USA

**Keywords:** breast cancer, diagnosis, LINC00310, oncogenic role, prognosis

## Abstract

Recent studies have revealed that long non‐coding RNAs (lncRNAs) are involved in different physiological processes and human diseases. However, to date, the function and overall clinical significance of the vast majority of lncRNAs in breast cancer remain largely unexplored. Here, we focused on LINC00310 by interrogating the breast invasive carcinoma data set of the Cancer Genome Atlas (TCGA). The results showed that LINC00310 was increased as breast cancer progressed, and the deregulation of LINC00310 was significantly associated with patients’ survival. Experiments with knockout (KO) approach by CRISPR/Cas9 system and the subsequent rescue experiments revealed that LINC00310 promoted cell proliferation by regulating c‐Myc expression in vitro. Nude mouse xenograft assay demonstrated that LINC00310 KO significantly suppressed tumour growth in vivo. Furthermore, we found that serum LINC00310 expression was significantly up‐regulated in patients with breast cancer, and receiver operating characteristic (ROC) curve analysis indicated that LINC00310 had a powerful capability of distinguishing patients with breast cancer from healthy individuals (the area under curve 0.828). Taken together, these results provide a more intuitive approach to explore the clinical relevance and functional roles of lncRNAs. As a result, lncRNAs, such as LINC00310, may be used in clinical applications as circulating markers for breast cancer.

## 1 INTRODUCTION

Breast cancer is the most common malignancy and the second leading cause of cancer‐related death in women worldwide.^1^ The age‐standardized incidence and mortality of breast cancer show a significant upward trend in China, and breast cancer alone is expected to account for 15% of all new cancer cases in women in 2015.^2^ Over the past decades, remarkable progress has been made in understanding the molecular mechanisms of breast cancer, and new personalized treatments have been developed. However, breast cancer is considered as a complex disease with high heterogeneity, exhibiting wide variability in prognostic pattern and treatment response.^3^, ^4^, ^5^, ^6^ Therefore, it is urgently necessary to identify novel biomarkers that may not only predict the biological behaviour and clinical outcome, but also improve design of treatment protocols and development of novel therapeutic candidates for breast cancer.

Long non‐coding RNAs (lncRNAs) are operationally defined as RNA molecules longer than 200 nucleotides without protein‐coding potential. They are proposed to constitute major proportion of human cellular transcripts.^7^, ^8^ Once considered to be transcriptional noise, lncRNAs are less well characterized than small non‐coding microRNAs.^9^, ^10^, ^11^ However, increasing evidence suggests that lncRNAs play important roles in various processes and provide a new insight into cancer biology.^12^, ^13^, ^14^ Like protein‐coding genes, lncRNAs can function as oncogenic or tumour suppressor genes and regulate target gene expression at multiple levels, including transcriptional and post‐transcriptional levels,^15^ thus affecting one or more of the cancer hallmarks, such as cell growth, apoptosis, differentiation, invasion and metastasis.^16^, ^17^ Abnormally expressed lncRNAs have been observed in various types of cancers, including breast cancer.^18^, ^19^ For instance, lncRNA HOTAIR (Hox transcript antisense intergenic RNA) is overexpressed in patients with breast cancer, and its deregulation is correlated with enhanced breast cancer metastasis.^20^ Up‐regulation of lncRNA BC200 contributes to breast cancer pathogenesis and progression by restraining apoptotic cell death due to its ability to regulate Bcl‐xL expression.^21^ However, the function and overall clinical significance of the vast majority of lncRNAs in breast cancer remain largely undetermined. Moreover, recent studies have demonstrated that lncRNAs exhibit greater expression restriction, and several lncRNAs have shown the potential as biomarkers for cancer diagnosis and prognosis.^22^, ^23^ Thus, there is an urgent need to explore functional roles of novel lncRNAs in breast cancer, which may provide a new molecular option for its diagnosis and prognosis.

In this study, LINC00310 was identified to be associated with prognosis of patients with breast cancer by interrogating the breast invasive carcinoma data set of the Cancer Genome Atlas (TCGA) at cBioPortal. We then characterized LINC00310 by knockout (KO) cell models and revealed that LINC00310 played an oncogenic role in breast cancer both in vitro and in vivo. Furthermore, we detected the expression of LINC00310 in serum samples of patients with breast cancer and evaluated its clinical significance as a potential biomarker for breast cancer diagnosis.

## 2 MATERIALS AND METHODS

### 2.1 Reagents

Primary antibodies used in this study were obtained from different sources as follows: c‐Myc was purchased from Abcam (Cambridge, MA, USA), and α‐tubulin and GAPDH were obtained from Protein Tech Group (Chicago, IL, USA). Secondary antibodies conjugated with IRDye 800CW or IRDye 680 were supplied by LI‐COR Biosciences (Lincoln, NE, USA). Breast cancer tissue cDNA arrays were purchased from OriGene (Rockville, MD, USA). PCR primers were synthesized by IDT (Coralville, IA, USA) and Shanghai Boshang Biological Company (Shanghai, China).

### 2.2 Cell lines

Human MCF‐7, MDA‐MB‐231, HMLE and HEK‐293T cells were obtained from American Type Culture Collection (Manassas, VA, USA). LM‐4142 cells were kindly provided by Dr. Joan Massagué (Memorial Sloan‐Kettering Cancer Center), which were originally derived from MDA‐MB‐231 cells as previously described.^24^ Breast cancer cells MCF‐7, MDA‐MB‐231 and LM‐4142 were maintained in RPMI‐1640 medium (Lonza, Walkersville, MD, USA) supplemented with 10% foetal bovine serum (FBS, Sigma‐Aldrich, St. Louis, MO, USA) and 2 mmol/L glutamine. Immortalized human mammary epithelial cells (HMLE) were cultured in DMEM/F12K supplemented with growth factor hEGF, hydrocortisone and insulin, while HEK‐293T cells were grown in DMEM containing 10% FBS and 2 mmol/L glutamine. Additionally, all culture media contained 100 U/mL penicillin and 100 μg/mL streptomycin (Lonza). Cells were incubated under standard conditions in a humidified chamber containing 5% CO_2_ at 37°C.

### 2.3 Patients and sample collection

A total of 95 subjects, including 48 patients with breast cancer and 47 healthy individuals, from Qilu Hospital of Shandong University between March 2017 and August 2017 were enrolled in this study. None of the patients received preoperative adjuvant therapy, and all these healthy subjects showed no evidence of disease. Serum samples were collected and separated within 2 hours following a two‐step centrifugation protocol (1500 ***g*** for 10 minutes at 4°C and 13 800 ***g*** for 15 minutes at 4°C) to completely remove the cell debris. Each supernatant was transferred into RNase‐ and DNase‐free tubes and stored at −80°C prior to further analysis. This study was approved by the Ethics Committee of Qilu Hospital of Shandong University, and informed consent was obtained from each participant.

### 2.4 Plasmid construction

Respective DNA fragments were amplified by PCRs for cloning purpose using Phusion enzyme from Thermo Fisher Scientific (Pittsburgh, PA, USA). Dual gRNAs targeting LINC00310 were designed based on CHOPCHOP (https://chopchop.rc.fas.harvard.edu/), and their sequences were listed in Table S1. For dual gRNA and the corresponding donor cloning, the same method recently developed in our laboratory was used.^25^ A donor vector carrying left and right arms homologous to the flanking regions of the targeting sites was generated to facilitate the selection of complete KO clones. PCR was performed with primer sets LINC00310‐left‐Spe I‐5.1 and LINC00310‐left‐Spe I‐3.1 (left arm), and LINC00310‐right‐Sal I‐5.1 and LINC00310‐right‐Sal I‐3.1 (right arm), while human genomic DNA was used as a template. These two fragments were sequentially cloned into donor vector carrying marker genes (GFP and PU, the puromycin resistance gene) at Spe I and Sal I sites. To generate LINC00310 expression construct for rescue experiments, the entire LINC00310 sequence was amplified by PCR using primers LINC00310‐R1‐5.1 and LINC00310‐Not1‐3.1 and then cloned into pCDH‐MSCV‐EF1‐GFP‐T2A‐Pu (System Bioscience). All amplified fragments were verified by DNA sequencing. All the primers used in plasmid construction were listed in Table S1.

### 2.5 Knockout of LINC00310 by CRISPR/Cas9

We used a dual gRNA approach to knock out LINC00310 by CRISPR/Cas9 system.^25^, ^26^ A donor vector carrying EF1‐GFP‐T2A‐PU flanked by LoxP and left or right arm derived from the outside regions of the targeting sites of LINC00310 was used to elevate the frequency of generating KO clones. The dual gRNA construct carrying Cas9 and donor vector were introduced into LM‐4142 cells by transient cotransfection, while the empty dual gRNA vector served as a control. One week later, the transfected cells were subjected to puromycin selection (1 μg/mL) for another 1 week. The surviving cells were sorted by FACS based on GFP signal into 96‐well plates, incubated for 1‐2 weeks and then expanded in 24‐well plates. Initial identification of KO clones was performed by genomic PCR, and potential clones were further verified by quantitative real‐time PCR (qRT‐PCR) as previously described.^25^

### 2.6 MTT assay

MTT assay was conducted to check the effect of LINC00310 on cell growth as previously described.^27^ Briefly, LM‐4142 cells were seeded into 96‐well plates at a density of 1000 cells/well, and the measurement was performed for 0‐4 days after seeding.

### 2.7 Clonogenic assay

To assess the colony‐forming ability of the cells, LM‐4142 cells were seeded into 6‐well plates at a density of 1000 cells/well. After 10 days, colonies were fixed and stained with 0.1% crystal violet before counting.

### 2.8 RNA isolation, RT‐PCR and qRT‐PCR

For cell lines, total RNA was isolated using Direct‐zol™ RNA MiniPrep kits (Zymo Research, Irvine, CA, USA) according to the manufacturer's instructions, and 0.5 μg RNA was reversely transcribed into complementary DNA (cDNA) by RevertAid™ Reverse Transcriptase (Fisher Scientific, Pittsburgh, PA, USA) with random primers (New England Biolabs, Ipswich, MA, USA) in a 10 μL reaction system. For serum samples, total RNA was isolated using TRIzol LS (Invitrogen, Carlsbad, CA) following the manufacturer's protocol, and reverse transcription was conducted using the Prime Script™ RT Reagent Kit (Takara, Dalian, Liaoning). The resultant cDNA then served as template for qRT‐PCRs. The expressions of LINC00310 and c‐Myc were specifically detected using the SYBR Green method with primers listed in Table S1. GAPDH or β‐actin was used as an internal control. Delta‐delta Ct values were used to calculate their relative expressions as previously described.^28^

### 2.9 Western blot analysis

Cells were harvested, and proteins were extracted from cells and quantified using Bio‐Rad Protein Assay Dye Reagent (Bio‐Rad, Hercules, CA, USA). Western blot analysis was performed as previously described.^29^

### 2.10 Animal work

The animal studies were carried out in accordance with animal use guidelines of National Institutes of Health, and the experimental protocol was approved by the University of Mississippi Medical Center (UMMC's) Animal Care and Use Committee. Animal work to determine the role of LINC00310 in tumour growth was performed according to the procedures as previously described.^30^ Briefly, female nude (nu/nu) mice (4‐5 weeks old) were purchased from Charles River (Wilmington, MA, USA). LM‐4142 cells with vector control or LINC00310 KO at the exponential phase were harvested, mixed with 50% Matrigel (BD Biosciences, San Jose, CA, USA) and then injected into the mammary fat pad at a dose of 1 × 10^6^ cells per spot. Eight days after injection, tumour growth was monitored every other day by measuring the tumour length (L) and width (W), and tumour volume was calculated by the formula as follows: volume = 1/2(L × W^2^).

### 2.11 Statistical analysis

Statistical significance between groups was analysed by Student's *t* test or Mann‐Whitney *U* test as appropriate. Receiver operating characteristic (ROC) curve was established for discriminating patients with breast cancer and controls using MedCalc 15.2.2 (MedCalc, Mariakerke, Belgium). Area under the ROC curve (AUC) was used to evaluate the diagnostic performance of serum LINC00310. Statistical analyses and graphing were conducted using SPSS version 22.0 and GraphPad Prism software. All *P* values were two‐sided, and *P *<* *.05 was considered as statistically significant.

## 3 RESULTS

### 3.1 LINC00310 is associated with the progression and survival outcome of breast cancer

**Figure 1:**
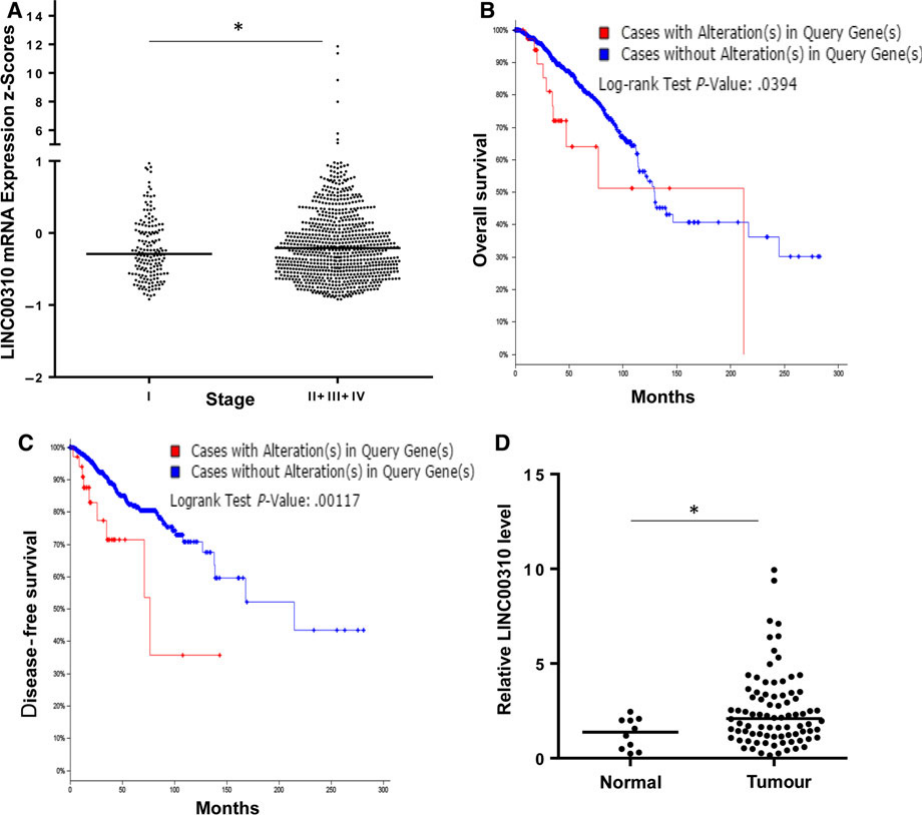
Association of LINC00310 expression with tumour stages and survival outcome. A, Expression level of LINC00310 in different stages of breast cancer. B,C, Kaplan‐Meier curve for overall survival (B) or disease‐free survival (C) based on alterations of LINC00310. D, LINC00310 expression in the OriGene breast cancer tissue cDNA array using qRT‐PCR assay. *P < .05

As deep sequencing becomes a commonly used tool to provide more valuable information, the expression data (RNA‐Seq) of LINC00310 were searched in breast cancer based on the Onco Query Language criteria “EXP ≥ 2” from the Cancer Genome Atlas (TCGA) data set at the cBioPortal for Cancer Genomics. We found that the alteration of LINC00310 with RNA expression occurred at a frequency of 4% in total of 1110 samples (Figure S1). The expression of LINC00310 was significantly higher in patients with advanced breast cancer compared with the patients with stage I breast cancer (Figure 1A), indicating that it might be involved in the progression of breast cancer. Next, we further determined whether deregulation of LINC00310 could predict clinical outcome of patients. We found that the up‐regulation of LINC00310 was significantly associated with patients’ survival (*P *<* *.05) (Figure 1B,C). Especially, patients with high LINC00310 expression had a significantly poorer disease‐free survival compared with those with low LINC00310 expression (Figure 1C). In addition, we profiled breast cancer cDNA arrays from OriGene by qRT‐PCR, and the results showed that the LINC00310 expression was elevated in breast cancer tissues compared with the normal tissues (Figure 1D). Taken together, these findings suggest that LINC00310 may play an oncogenic role and can be explored as a prognostic biomarker for breast cancer.

### 3.2 Generation of LINC00310 KO with CRISPR/Cas9 system using a dual gRNA approach

**Figure 2:**
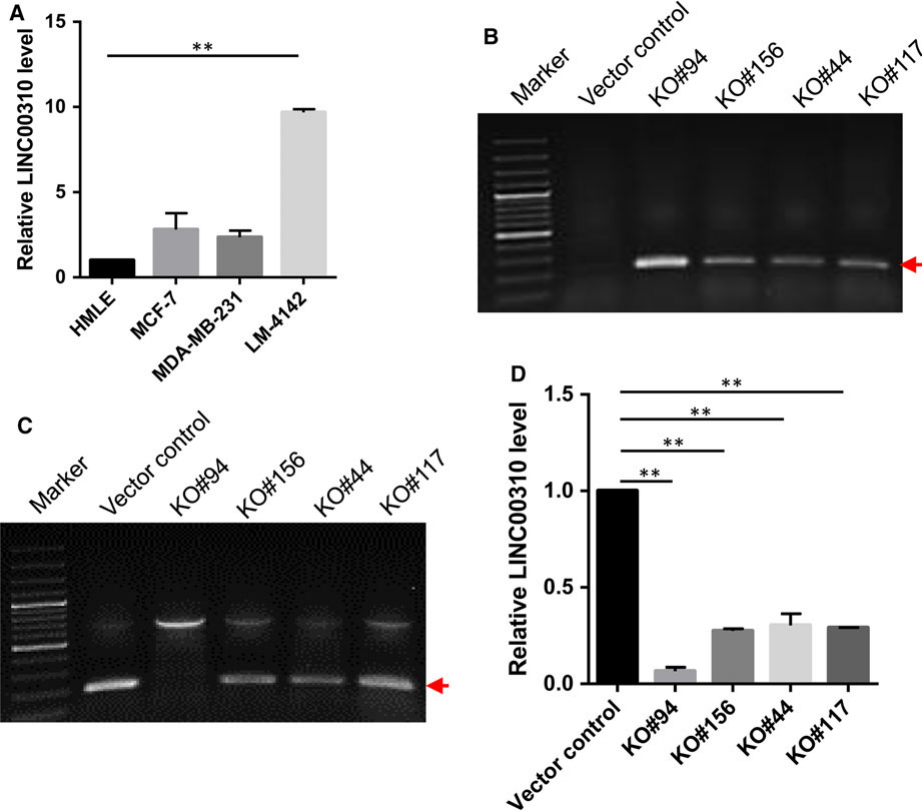
Generation of LINC00310 KO in LM‐4142 cells with CRISPR/Cas9 system using a dual gRNA approach. A, LINC00310 was up‐regulated in breast cancer cell line LM‐4142 compared with the non‐malignant breast cells (HMLE). B, Detection of LINC00310 deletion by genomic PCR using primers LINC00310‐outside‐5.1 and LINC00310‐outside‐3.1. The red arrow indicates deletion bands. C, Detection of LINC00310 target region using genomic DNA as a template and primers LINC00310‐RT‐5.1 and LINC00310‐RT‐3.1. The red arrow indicates inside bands of the targeting sites. D, Expression of LINC00310 in KO cells compared with the vector control by qRT‐PCR. **P < .01

To better characterize the oncogenic role of LINC00310, we further checked its expression in different cell lines and found that LINC00310 was up‐regulated in breast cancer cell line LM‐4142 compared with the non‐malignant HMLE cells (Figure 2A). Therefore, we took advantage of a dual gRNA approach with CRISPR/Cas9 system to knock out LINC00310 in LM‐4142 cells. These two gRNAs were located at the 5ʹ and 3ʹ extremities of the LINC00310 gene, enabling us to delete the entire gene (Figure S2). To identify KO clones of LINC00310, we initially screened hundreds of randomly picked single colonies by genomic PCR. Firstly, we amplified the dual gRNA–targeted region using primers derived from outside of two gRNAs (Figure S2) and four (#94, #156, #44 and #117) colonies showed deletion bands (Figure 2B). Genomic PCR using primers inside of the targeting sites was then carried out. As shown in Figure 2C, it is evident that no PCR product was detected in KO #94, suggesting that it was a complete KO clone. However, there were still inside bands in the other three clones (#156, #44 and #117), indicating that they were all partially KO clones. Consistent with these results, although the sensitive qRT‐PCR caused some background amplification, we found that the LINC00310 expression was substantially decreased (Figure 2D). Finally, we selected two clones (#94 and #44) for further characterization.

### 3.3 LINC00310 KO inhibits tumour cell growth and c‐Myc expression in vitro

**Figure 3:**
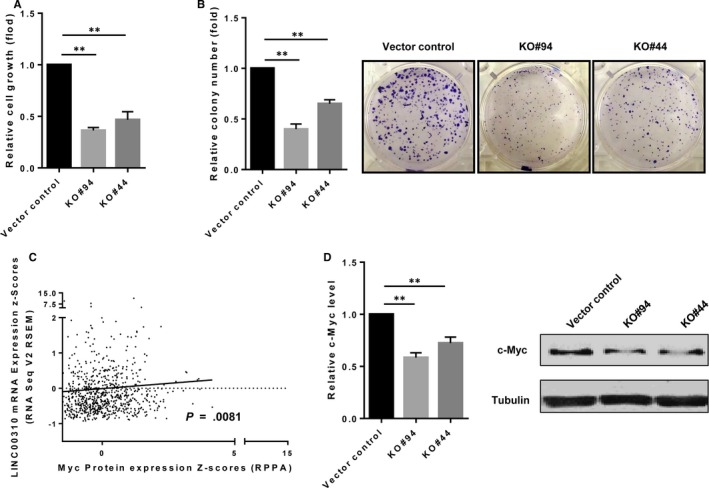
LINC00310 KO suppresses tumour cell growth and c‐Myc expression in vitro. A, Effect of LINC00310 on cell growth as measured by the MTT assay. B, LINC00310 KO suppressed cell growth as detected by colony formation assays. C, The correlation between Myc protein expression and LINC00310 level in breast cancer was analysed using Spearman correlation analysis. D, LINC00310 KO suppressed c‐Myc expression at the mRNA level. E, LINC00310 KO suppressed c‐Myc expression at the protein level. Error bars represent SEM, **P <* *.01

We next determined the biological consequence of LINC00310 ablation in tumorigenesis. As expected, MTT assays showed that LINC00310 KO significantly reduced the cell growth (Figure 3A). Meanwhile, we found that the number of colonies was much smaller in LINC00310 KO cells compared with the vector control as detected by clonogenic assays (Figure 3B). These results provide evidence that LINC00310 affects tumour cell growth and proliferation, and thus, LINC00310 may serve as a potential oncogene.

To demonstrate how LINC00310 impacts cell growth and proliferation, we attempted to analyse a number of genes such as Myc, which play essential roles in breast tumorigenesis using TCGA data set from the cBioPortal for Cancer Genomics. Spearman correlation analysis revealed that the Myc protein expression was significantly associated with LINC00310 level in breast cancer (*P *=* *.0081) (Figure 3C). Therefore, we identified the effect of LINC00310 on c‐Myc expression. As shown in Figure 3D, qRT‐PCR data showed that LINC00310 KO decreased the c‐Myc mRNA expression. At the protein level, we also observed a significant inhibition of c‐Myc in both LINC00310 KO clones (Figure 3E). These results suggest that LINC00310 may impact tumour cell growth and proliferation by regulating c‐Myc expression.

### 3.4 LINC00310 KO inhibits tumour growth and c‐Myc expression in vivo

**Figure 4:**
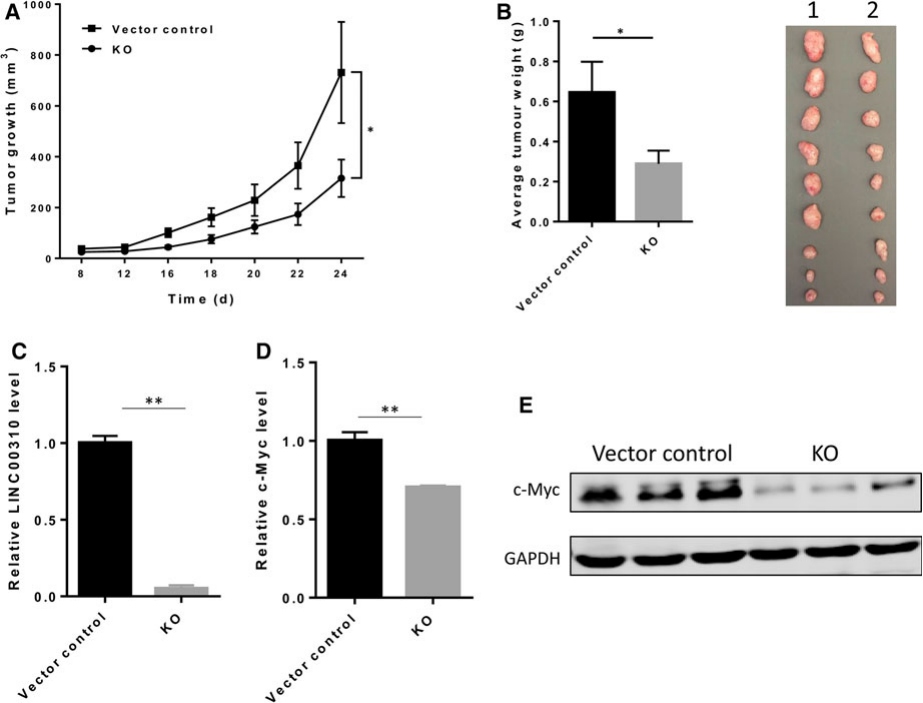
LINC00310 KO suppresses tumour growth and c‐Myc expression in vivo. A, Tumour growth for vector control and LINC00310 KO in nude mice. B, LINC00310 KO decreased tumour weight. Actual tumour size after harvest was shown in the right panel (1, Vector control; 2, KO). C, Detection of LINC00310 level in xenograft tumours. D, LINC00310 KO suppressed c‐Myc expression at the mRNA level in xenograft tumours. E, LINC00310 KO suppressed c‐Myc expression at the protein level in xenograft tumours. Three tumours were randomly selected for RNA or protein extraction in (C,D,E). *P <* *.05 and **P <* *.01

We then checked the effect of LINC00310 ablation on primary tumour growth using nude mouse xenograft models. The complete KO clone (KO #94) and vector control cells were selected to inject into the mammary fat pads of female nude mice. In consistent with in vitro results, we found that LINC00310 KO inhibited tumour growth compared with the vector control (Figure 4A). Moreover, the LINC00310 KO also decreased tumour weight (Figure 4B, left). There was an obvious visual difference in the tumour size between the two groups (Figure 4B, right), suggesting the role of LINC00310 in primary tumour growth of breast cancer. As expected, we found little expression of LINC00310 in LINC00310 KO tumours (Figure 4C). Tumours derived from LINC00310 KO exhibited lower c‐Myc expression at both the mRNA (Figure 4D) and protein levels (Figure 4E) than those derived from vector control, further indicating that LINC00310 played a critical role in tumour growth through regulation of c‐Myc.

### 3.5 Re‐expression of LINC00310 restores tumour growth and c‐Myc expression

**Figure 5:**
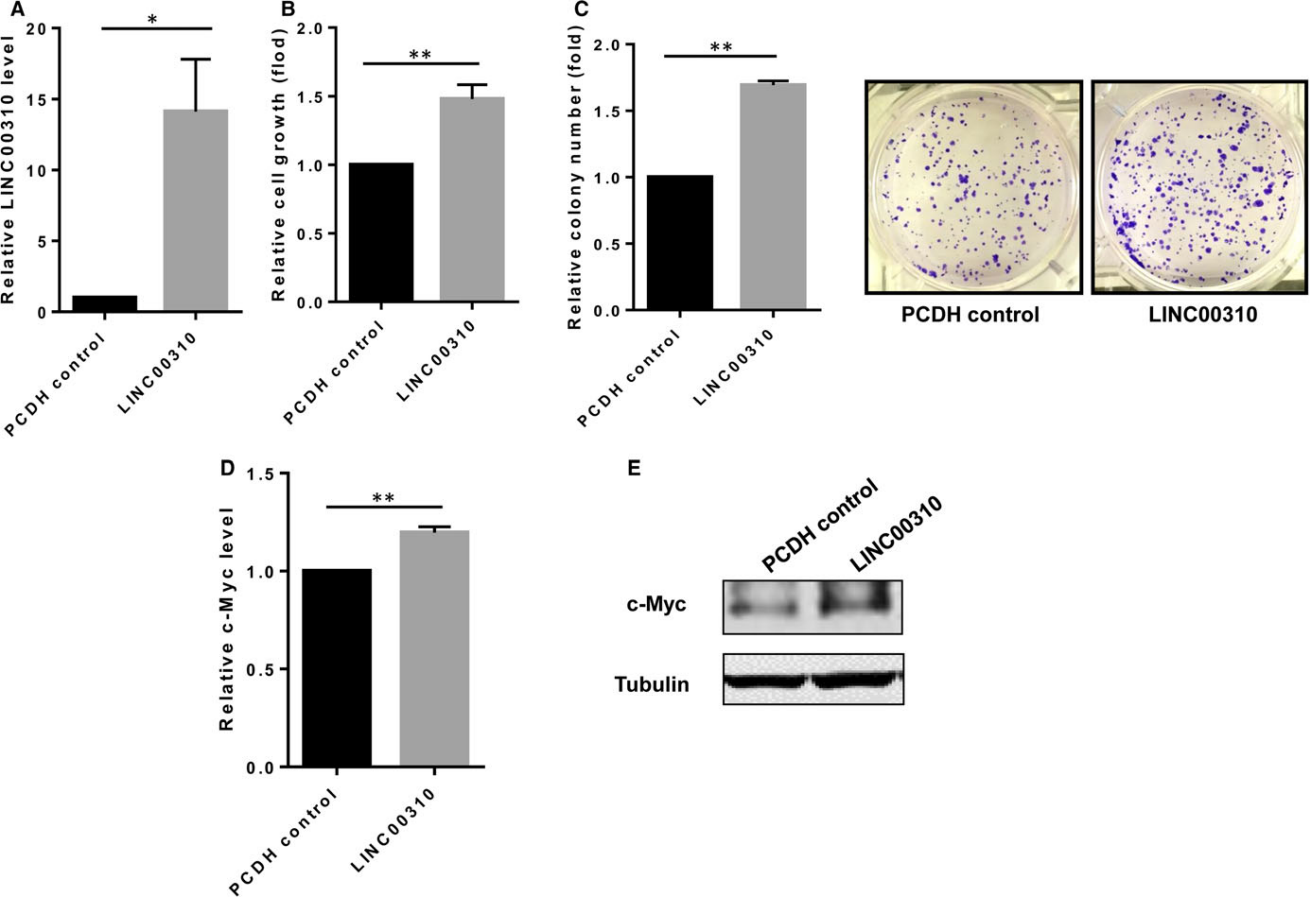
Re‐expression of LINC00310 in the KO cells (LINC00310) restores tumour growth and c‐Myc expression compared with the empty vector control (PCDH control). A, Re‐expression of LINC00310 in the KO#94 cells restored LINC00310 expression as detected by qRT‐PCR. B, Re‐expression of LINC00310 in KO #94 cells increased tumour cell growth as measured by the MTT assay. C, Re‐expression of LINC00310 in KO #94 cells enhanced cell growth as detected by colony formation assays. D, Re‐expression of LINC00310 in KO #94 cells increased c‐Myc expression at the mRNA level as measured by qRT‐PCR. E, Re‐expression of LINC00310 in KO #94 cells significantly increased c‐Myc expression at the protein level as determined by Western blot analysis. *P <* *.05 and **P <* *.01

To further confirm whether the growth inhibition was caused by the c‐Myc down‐regulation which was regulated by LINC00310 KO, we performed the rescue experiments to re‐express LINC00310 in complete KO clone (KO #94) (Figure 5A). As expected, MTT assays showed that re‐expression of LINC00310 in the KO cells significantly increased the cell growth (Figure 5B). Consistent with this result, the number of colonies was restored by LINC00310 re‐expression compared with the vector control as detected by clonogenic assays (Figure 5C). Importantly, ectopic expression of LINC00310 increased the c‐Myc expression at both the mRNA and protein levels (Figure 5D,E). Taken together, these findings further indicate that LINC00310 may regulate the c‐Myc expression, contributing to its oncogenic role.

### 3.6 Diagnostic performance of serum LINC00310 expression

**Figure 6:**
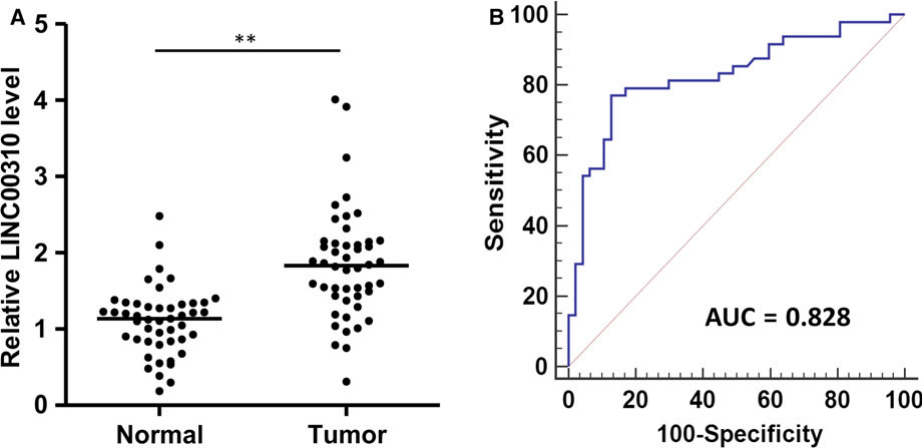
Diagnostic performance of serum LINC00310 expression. A, Relative expression analysis of LINC00310 in serum of patients with breast cancer (tumour) and healthy controls (Normal) using qRT‐PCR. B, ROC curve analysis for the detection of breast cancer using serum LINC00310. **P < .01

Given that LINC00310 might play an oncogenic role in breast cancer, we tried to evaluate whether LINC00310 could be used as a potential biomarker for diagnosis of breast cancer. We determined the expression of LINC00310 by qRT‐PCR, using 95 serum samples, including 48 patients with breast cancer and 47 healthy controls. The results showed that serum LINC00310 expression in patients with breast cancer was significantly up‐regulated in comparison with healthy controls (Figure 6A). An ROC curve was constructed based on the above findings to assess the diagnostic value of LINC00310. The results demonstrated that LINC00310 had a strong capability of distinguishing patients with breast cancer from healthy individuals, with an AUC of 0.828 (95% confidence interval [CI] = 0.737‐0.898) (Figure 6B). When the cut‐off value was set to the optimal point (1.402), the sensitivity and specificity were 77.08% and 87.23%, respectively. These findings suggest that serum LINC00310 possesses strong diagnostic power and can be used as a potential biomarker for the diagnosis of breast cancer.

## 4 DISCUSSION

Accumulating evidence has demonstrated that lncRNAs are dysregulated in many different cancers, and the expression patterns of lncRNAs exhibit greater tissue specificity compared with protein‐coding genes.^16^, ^31^, ^32^ The functions of lncRNAs are complicated because they can positively/negatively regulate multiple targets by binding DNA, RNA or proteins at both transcriptional and post‐transcriptional levels.^33^, ^34^, ^35^ Therefore, it is definitely worthwhile to further investigate their potential roles and clinical utility in cancer progression.

The development of high‐throughput sequencing technologies offers the opportunities to explore a wealth of novel, unannotated transcripts, especially long non‐coding transcripts.^36^, ^37^ Compared with protein‐coding genes, lncRNAs are found to constitute a much larger proportion,^38^ which may serve as a rich source of potential biomarkers. It is often a challenge for researchers to focus on lncRNAs with biological functions and clinical potentials due to the overwhelming amount of lncRNAs. In our previous study, we have identified that the up‐regulation of a lncRNA signature consisting of nine lncRNAs (LINC00705, LINC00310, LINC00704, LINC00574, FAM74A3, UMODL1‐AS1, ARRDC1‐AS1, HAR1A and LINC00323) can predict recurrence of breast cancer.^39^ Therefore, we focused on LINC00310 in the present study. We attempted to further explore the clinical attributes, prognostic value and potential targets of LINC00310 on an open platform, which offered a good starting point for further experimental confirmation. This study provides important clues for researchers with little bioinformatics knowledge to conduct lncRNA study using the existing public data. Compared with RNA‐Seq or gene microarray analysis, lncRNA databases, such as the cBioPortal, can serve as a more convenient and effective approach to investigate the relationship between lncRNAs and diseases before launching real experiments.

Breast cancer is one of the top killers in women health. Given that the disease in early stages often appears with non‐specific symptoms, advanced or terminal diagnosis may occur by the time symptoms develop, with poor prognosis and poor treatment effect. Thus, there remains a considerable need for identification of novel biomarkers and better understanding of the molecular mechanisms underlying breast cancer. So far, several lncRNAs have been reported to show different expression patterns and be closely associated with breast cancer.^40^ For example, HOTAIR expression is increased in both primary breast tumours and metastases and can significantly predict subsequent metastasis and death of breast cancer.^20^ Moreover, researchers have revealed that HOTAIR is associated with metastasis in patients with ER‐positive breast cancer, which may serve as an independent marker.^41^ Another example includes LINC00472, which is regulated by promoter methylation and related to disease‐free survival in grade 2 patients with breast cancer.^42^ In addition, recent studies have assessed the association between lncRNA signature and breast cancer subtypes as well as patients’ survival.^39^, ^43^ To the best of our knowledge, no study has explored the clinical application and function of LINC00310 in breast cancer to date. Given that lncRNAs may be involved in cancer progression, we first explored whether LINC00310 expression could be a predictor in the clinical management of breast cancer. We found that LINC00310 expression was increased as tumours progressed. Its expression in patients with advanced breast cancer was significantly higher compared with those with stage I breast cancer. Moreover, we revealed that altered expression of LINC00310 was significantly associated with survival of patients with breast cancer. These findings provide evidence that LINC00310 may function as an oncogene in breast cancer and can be considered as a potential prognostic factor.

In support of this notion, we further confirmed the oncogenic role of LINC00310 by experiments in vitro and in vivo. For example, LINC00310 was up‐regulated in breast cancer specimens. LINC00310 ablation suppressed tumour cell growth both in vitro and in vivo. Moreover, LINC00310 KO mice exhibited much less tumour growth and overall tumour weight. Our further database analysis suggested the association between LINC00310 and Myc. It is well known that c‐Myc, as a proto‐oncogene, can affect numerous cellular processes to favour malignant transformation by regulating the expression of many target genes.^44^, ^45^ Various mechanisms, including amplification, transcriptional activation or post‐transcriptional regulation, may contribute to the c‐Myc dysregulation. Considering the effect of LINC00310 on c‐Myc expression in the present study, we provide evidence that alteration of c‐Myc by LINC00310 may induce the LINC00310‐mediated tumorigenesis. In addition, we found that the protein expression of CDK4 was also decreased in LINC00310 KO cells both in vitro and in vivo (Figure S3), further suggesting that LINC00310 may promote cell proliferation by regulating cell cycle. However, only one cell line was used in this study to conduct KO and rescue experiments, and the detailed mechanism remained undetermined. In particular, further studies are needed to reveal the complex regulatory network between LINC00310, c‐Myc and CDK4, which may assist not only in the understanding of underlying mechanisms of the lncRNA‐mediated tumorigenesis, but also in the identification of novel therapeutic targets for breast cancer.

Recently, circulating RNAs in blood have been an emerging field for non‐invasive diagnostic applications.^46^ Several studies have suggested that circulating lncRNAs can be protected from endogenous RNase activity, making them highly stable and suitable as markers of the non‐invasive analysis for patient samples.^47^, ^48^ In the present study, we found that the LINC00310 expression in serum of patients with breast cancer was significantly up‐regulated compared with the healthy controls. ROC curve analysis demonstrated that LINC00310 had a powerful capability of distinguishing patients with breast cancer from healthy individuals (Figure 6). These findings underscore the potential significance of LINC00310 in serving as a biomarker for the diagnosis of breast cancer. In this regard, our study was consistent with recent reports on lncRNAs as potential circulating biomarkers for breast cancer. For example, plasma lncRNA GAS5 is significantly decreased in the postoperative samples compared with paired preoperative samples, indicating that GAS5 may have the potential to assess the surgical effects for breast cancer.^49^ Of interest, circulating DNA has been found to be the major form of HOTAIR‐derived fragment in serum, and circulating HOTAIR DNA may be a potential marker for distinguishing breast cancer patients from healthy individuals.^50^ Taken together, these findings may generate a great enthusiasm in exploring the potential of circulating lncRNAs as minimally invasive markers. However, as these studies generally focus on circulating individual cancer‐specific lncRNA as cancer biomarkers, a caveat may be that a single lncRNA may not be sufficient to serve as a reliable biomarker due to the complex pathophysiology of cancer initiation and progression. Therefore, simultaneous evaluation of tumour‐associated circulating lncRNA panel may increase the sensitivity and specificity of diagnosis and prognosis in breast cancer. Towards this direction, one recent study using a small cohort has demonstrated that a three‐lncRNA signature, including ANRIL, HIF1A‐AS2 and UCA1, can serve as diagnostic markers for differentiating between triple‐negative and non‐triple‐negative breast cancer.^51^ Evidently, further systematic and multicenter studies of larger independent samples are urgently needed to develop the possible clinical applications of circulating lncRNAs in patients with breast cancer.

In summary, our results suggested that LINC00310, as an oncogene, could be considered as a potential candidate for prognostic evaluation of breast cancer, and it might promote tumorigenesis by regulating c‐Myc expression. Moreover, we demonstrated that serum LINC00310 possessed strong diagnostic power and could be used as a potential biomarker for the diagnosis of breast cancer. Although further studies are needed to reveal the detailed mechanism of LINC00310‐mediated breast cancer progression and confirm the diagnostic value of serum LINC00310 with a larger number of subjects, our findings provide a more intuitive approach to predict the clinical relevance and explore their functional roles of lncRNAs. As a result, lncRNAs, such as LINC00310, may be used in clinical applications as circulating markers for breast cancer.

## CONFLICT OF INTEREST

The authors declare that they have no competing interests.

## AUTHOR CONTRIBUTION

JL conceived the study, performed experiments and wrote the manuscript. JL, WP and LD performed the experiments and analysed the data. QY assisted in collecting serum samples and analysing the data. CW and YM conceived this research and provided the financial support. YM supervised the study and revised the manuscript. All authors read and approved the final manuscript.
