# Supplementary figures and images for: The oncogenic potentials and diagnostic significance of long non‐coding RNA LINC00310 in breast cancer
Source: J Cell Mol Med. 2018 Jul 11;22(9):4486–95. doi: 10.1111/jcmm.13750 (PMC6111859; doi:10.1111/jcmm.13750)

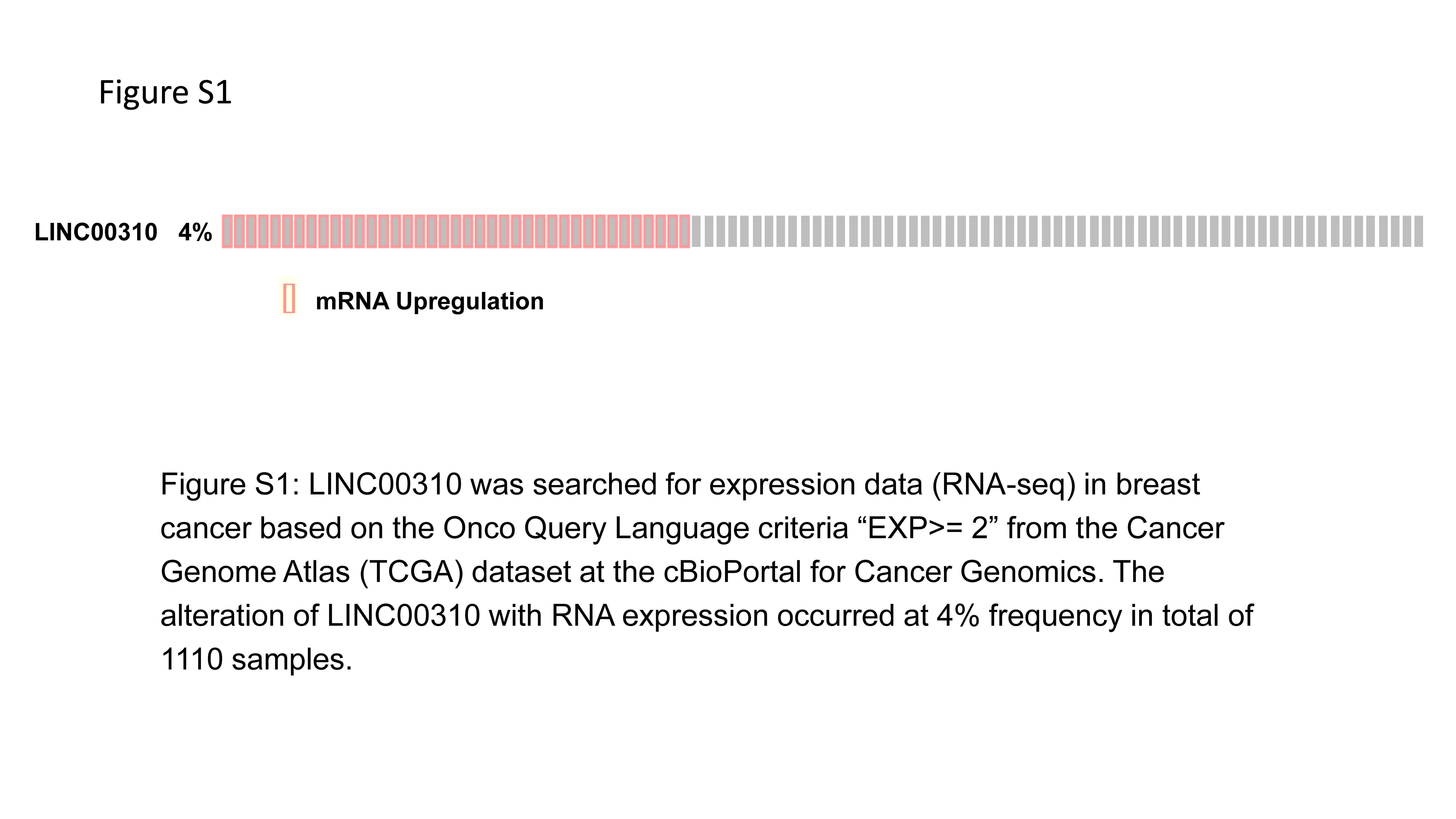

Supplement: Supplementary file 1 [file JCMM-22-4486-s001.tif]

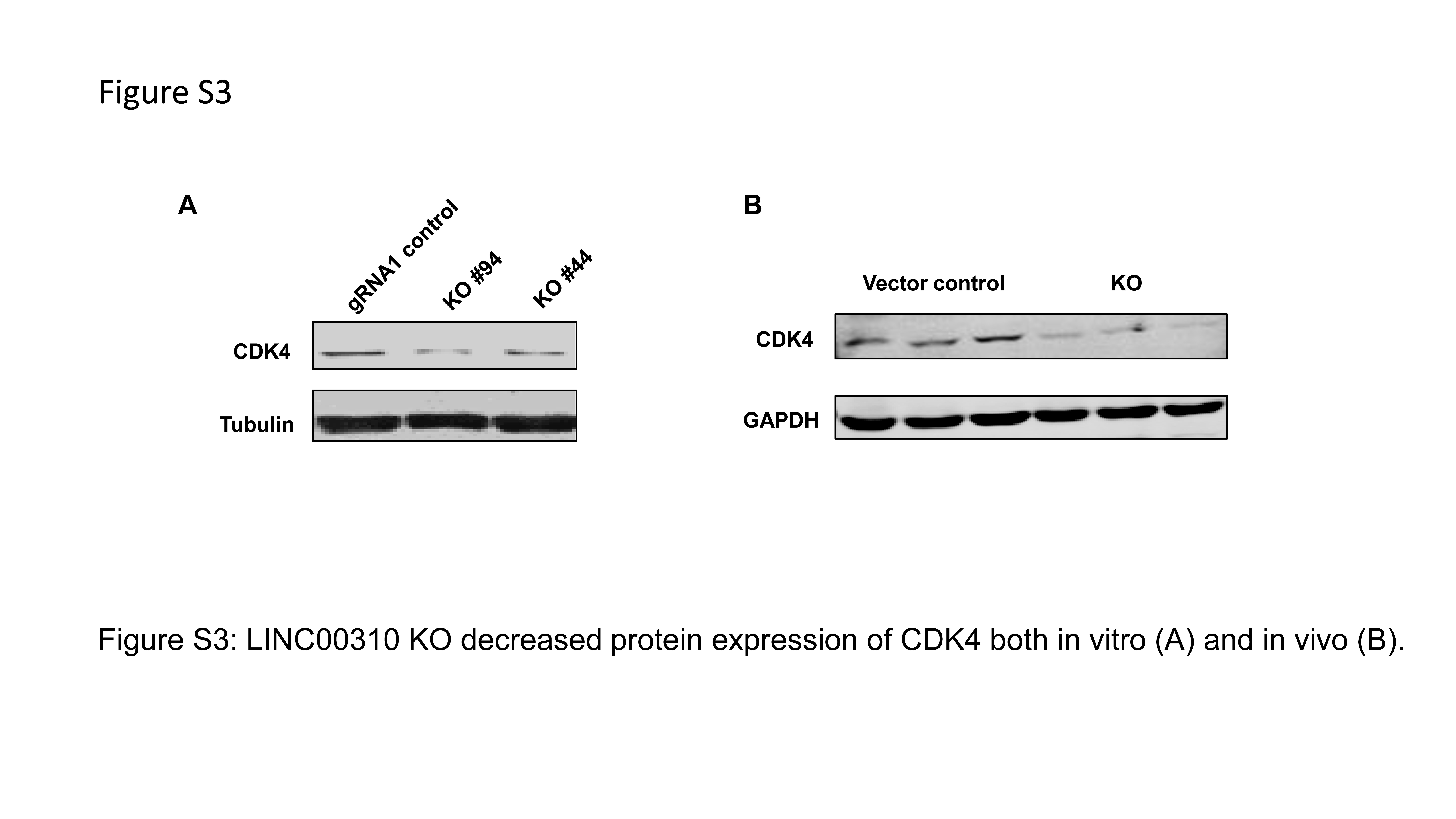

Supplement: Supplementary file 3 [file JCMM-22-4486-s003.tif]
