# Supplementary material for: The oncogenic potentials and diagnostic significance of long non‐coding RNA LINC00310 in breast cancer
Source: J Cell Mol Med. 2018 Jul 11;22(9):4486–95. doi: 10.1111/jcmm.13750 (PMC6111859; doi:10.1111/jcmm.13750)
Supplement: Supplementary file 4 [file JCMM-22-4486-s004.docx]

**Table S1: Primers used in this study**

| Primer | Sequence |
| --- | --- |
| For detection |  |
| c-Myc-RT-5.1 | CCTACCCTCTCAACGACAGC |
| c-Myc-RT-3.1 | CTCTGACCTTTTGCCAGGAG |
| LINC00310-RT-5.1 | TCTCTTGGGTCTTGCTTGCT |
| LINC00310-RT-3.1 | ACATGGGGATTATGGGGATT |
|  |  |
| For dual gRNA |  |
| LINC00310 T1 | GAGAGGAAAATGAAACACAG |
| LINC00310 T2 | AGGCAATCCTGTGAAGTAGA |
|  |  |
| For donor vector |  |
| LINC00310-left-Spe I-5.1 | GACGGCCAGTGAAACTAGTTAAGCAGGGATGAGGGCAGG |
| LINC00310-left-Spe I-3.1 | CCTGTACAGGTACCACTAGTATTAGGATAAATAGCTAATG |
| LINC00310-right-Sal I-5.1 | TCTTATCATGTCTGGTCGACTAGCAAATGAACACGCATGT |
| LINC00310-right-Sal I-3.1 | TTGATATCACCGGTGTCGACAATCAGGACGACACATCTTC |
|  |  |
| For cloning LINC00310 |  |
| LINC00310-R1-5.1 | TTCTAGAGCTAGCGAATTCGCAGATTCTTGGCAGACCTC |
| LINC00310-Not1-3.1 | TCGCAGATCCTTGCGGCCGCTATTTCCTGTCTCCAAATTA |
|  |  |
| For genomic PCR |  |
| LINC00310-outside-5.1 | TAGGTAAATGTGTGTCATGG |
| LINC00310-outside-3.1 | CACGCATGCGAGCATGTGTG |
